# Racial and Ethnic Disparities in Hospital Breastfeeding Care in the US

**DOI:** 10.1007/s10995-025-04065-y

**Published:** 2025-01-29

**Authors:** Jane Lazar Tucker, Kimberly Arcoleo, Diane DiTomasso, Brietta M. Oaks, Howard Cabral, Thaís São-João

**Affiliations:** 1 College of Nursing, University of Rhode Island, 350 Eddy St, Providence, RI 02903 USA; 2 College of Health Sciences, University of Rhode Island, 55 Lower College Rd, Kingston, RI 02881 USA; 3 Department of Biostatistics, Boston University School of Public Health, 801 Massachusetts Ave, 3rd floor, Boston, MA 02118 USA

**Keywords:** Racial groups, Ethnicity, Baby-friendly, Breast Feeding, PRAMS

## Abstract

**Objectives:**

This study examines the associations between race and ethnicity and receipt of Baby Friendly Hospital Initiative (BFHI) key clinical practices that support breastfeeding in US hospitals.

**Methods:**

National data from 2016 to 2019 CDC PRAMS were analyzed. Our sample included 60,395 mothers who initiated breastfeeding with healthy, term newborns. We conducted adjusted regression analyses to compare the odds of receiving individual key clinical practices that support breastfeeding, as well as the percent of key clinical practices received.

**Results:**

While some key clinical practices were received at high rates, less than 25% of mothers received 100% of recommended key clinical practices. Compared to White non-Hispanic mothers, mothers from various racial and ethnic groups were at lower odds of receiving 100% of key clinical practices: Black non-Hispanic [adjusted odds ratio (AOR) 0.59, 95% confidence interval (CI) (0.47–0.65)], English-Speaking Hispanic [AOR 0.79, 95% CI (0.71–0.88)], Spanish-speaking Hispanic [AOR 0.63, 95% CI (0.53–0.73)], and Asian/Pacific Islander non-Hispanic [AOR 0.54, 95% CI (0.47–0.63)].

**Conclusions for Practice:**

Despite a steady increase in the number of BFHI hospitals in the US, there are racial and ethnic disparities in the receipt of BFHI key clinical practices. More US hospitals must adopt BFHI key clinical practices and consistently implement those practices for every racial and ethnic group.

**Supplementary Information:**

The online version contains supplementary material available at 10.1007/s10995-025-04065-y.

## Significance

What is Already Known on the Subject?

What this Study adds?

Researchers have demonstrated that racial and ethnic disparities may exist in receipt of BFHI key clinical practices, with Black non-Hispanic and Hispanic mothers receiving fewer clinical practices compared to White non-Hispanic mothers. These studies have been limited geographically, included only small sample sizes, and did not explore the experiences of various racial and ethnic groups.

In US hospitals, few women receive the recommended key clinical practices that support breastfeeding, and Black non-Hispanic, Hispanic (both English and Spanish-speaking), and Asian/Pacific Islander non-Hispanic are significantly less likely to receive high-quality breastfeeding care compared to White non-Hispanic women.

**Supplementary Information:**

The online version contains supplementary material available at 10.1007/s10995-025-04065-y.

## Introduction

Since the early 1990s, the Baby Friendly Hospital Initiative (BFHI) has resulted in a steady increase in the number of hospitals that adhere to the “Ten Steps to Successful Breastfeeding.” This bundle of evidence-based key clinical practices support breastfeeding and include practices such as skin-to-skin contact immediately after delivery and avoiding artificial milk substitutes unless medically indicated (Baby-Friendly, 2024). In 2019, more than 28% of births occurred in over 600 BFHI facilities across the United States (Baby-Friendly, 2024). Receipt of BFHI key clinical practices during the delivery hospital stay is an important determinant of whether a woman will breastfeed successfully (Barrera et al., 2019; Crenshaw & Budin, 2020; Kivlighan et al., 2020). Notably, the positive impact of hospital breastfeeding support on breastfeeding outcomes persists across racial and ethnic groups (Kivlighan et al., 2020; Sebastian et al., 2019).

Though rates of breastfeeding have improved in recent years across all racial and ethnic groups, disparities in breastfeeding outcomes persist among Black and Hispanic women compared to White women (Beauregard et al., 2019; Chiang et al., 2021). This occurs even though women from all racial and ethnic backgrounds report a high intention to breastfeed. Nine out of ten pregnant women state the intention to breastfeed, yet Black and Hispanic women are about 40% less likely to meet that goal compared to White women (Hamner et al., 2021). The gap observed between breastfeeding intention and breastfeeding engagement among women who are Black and Hispanic suggests that there are barriers to breastfeeding among some racial and ethnic groups that contribute to early breastfeeding cessation.

Given that BFHI key clinical practices influence breastfeeding success, the rate at which women from different racial and ethnic groups receive these practices must be assessed. In few prior studies have researchers examined the receipt of hospital practices that support breastfeeding by race and ethnicity, though preliminary research suggests that disparities exist. Studies that have been conducted were focused almost exclusively on Black women, were geographically limited, and/or had small sample sizes (Gee et al., 2012; Gross et al., 2015; Johnson et al., 2016; Munn et al., 2018; Sebastian et al., 2019). Only one prior study included Hispanic and American Indian women, and no studies were found that included Asian/Pacific Islander women (Sebastian et al., 2019).

The scarcity of what is known about discrepancies in breastfeeding care across racial and ethnic groups points to the need for further research with population-based samples, including all racial and ethnic groups, representing a larger geographic area in the US. To properly address these disparities, a comprehensive examination of the experiences of diverse populations is pivotal to better understand how women from different racial and ethnic groups experience hospital practices that support breastfeeding. Therefore, the aim of this study is to identify the extent to which maternal race and ethnicity are associated with a postpartum mother’s receipt of BFHI key clinical practices during the birth hospitalization.

## Methods

### Data

The research and reporting for this paper adhered to the STROBE (Strengthening the Reporting of Observational Studies in Epidemiology) guidelines (von Elm et al., 2014). We examined differences in receipt of evidence-based breastfeeding care by race and ethnicity using data from the 2016 to 2019 National Pregnancy Risk Assessment Measurement System (PRAMS) survey. Use of these data provided a unique opportunity to understand the breastfeeding experiences of women that are historically underrepresented in survey data. PRAMS is a surveillance project conducted by the Centers for Disease Control and Prevention (CDC) in partnership with state health departments, which collects population-based survey data on maternal experiences during and after pregnancy, including women’s experiences of breastfeeding care during their delivery hospital stay (CDC, 2022c). PRAMS has 51 sites, which represent approximately 83% of all US births (Shulman et al., 2018). Sites sample between 100 and 250 new mothers each month, with samples ranging between 1300 and 3400 women per site each year (CDC, 2022b). Mothers respond to the survey via mail or telephone call within 2–6 months after delivery (CDC, 2022b). PRAMS requires that the weighted percentage responding from each state be at least 50% in order for the data from that state to be included in the analytic files (CDC, 2022a). PRAMS participants are sampled from the birth certificate, which is used to derive nonresponse weights; when applied to the sample data, the results can be generalized to the entire population of births (CDC, 2022b). This study was designated exempt and informed consent was waived by the Institutional Review Board at the University of Rhode Island because the data were deidentified.

### Study Population

We included women with a full-term (37 weeks or greater), singleton, live birth in US hospitals. Since information on breastfeeding intention is unavailable in PRAMS, we used breastfeeding initiation as a proxy for intention. Mothers who responded “Yes” to the PRAMS question “Did you ever breastfeed or pump breast milk to feed your new baby, even for a short period of time?” were eligible for inclusion. We excluded respondents if there was no available information on race and ethnicity or if the infant was not living with mother at the time of the survey. See Supplementary Fig. 1 for detailed information on inclusion and exclusion criteria. After applying inclusion and exclusion criteria, 44 of the 51 total sites remained.

### Measures

Maternal race and ethnicity were self-reported on the birth certificate. We combined the race and ethnicity variables to categorize women as Hispanic, non-Hispanic White, non-Hispanic Black, non-Hispanic Asian and Pacific Islander, non-Hispanic American Indian and Alaska Native, and non-Hispanic Mixed Race. We further differentiated Hispanic women as Spanish-speaking or English-speaking since acculturation and nativity are essential predictors of breastfeeding outcomes among Hispanic women (Bigman et al., 2018; Eilers et al., 2020). PRAMS participants responded to the questionnaire in English or Spanish at sites where they were given the option. Mothers who self-identified as Hispanic on the birth certificate and chose to respond to the questionnaire in Spanish were categorized as Hispanic-Spanish Speaking; the remaining respondent mothers were categorized as Hispanic-English Speaking. Due to the small sample size, those categorized as “other” race or ethnicity were excluded from the analyses.

The outcome of interest, receipt of evidence-based breastfeeding care, was examined using a group of survey items on the PRAMS questionnaire related to the BFHI key clinical practices. In Table 1, the BFHI Ten Steps are mapped to the corresponding question(s) on the PRAMS survey. The first two steps relate to hospital policy and staff training and were not assessed by PRAMS. Steps 3–10 are the key clinical practices that represent evidence-based breastfeeding care. PRAMS participants indicated yes or no to whether they endorsed the item(s).

**Table 1:** Baby friendly hospital initiative ten steps to successful breastfeeding mapped to PRAMS survey items

The ten steps to successful breastfeeding	PRAMS Survey items (Yes/No)	“Ideal breastfeeding support”
CRITICAL MANAGEMENT PROCEDURES:		
1. Have a written breastfeeding policy that is routinely communicated to all healthcare staff.	*Not assessed by PRAMS*	
2. Train all healthcare staff in the sls necessary to implement this policy.	*Not assessed by PRAMS*	
KEY CLINICAL PRACTICES:		
3. Discuss the importance and management of breastfeeding with pregnant women and their families.	“Hospital staff gave me information about breastfeeding”	YES
4. Facilitate immediate and uninterrupted skin-to-skin contact and support mothers to initiate breastfeeding as soon as possible after birth.	“My baby was placed in skin-to-skin contact within the first hour of life”	YES *and*
	“I breastfed in the first hour after my baby was born”	YES
5. Support mothers to initiate and maintain breastfeeding and manage common difficulties.	“Hospital staff helped me learn to breastfeed”	YES
6. Do not provide breastfed newborns any food or fluids other than breast-milk, unless medically indicated.	“My baby was fed only breastmilk at the hospital”	YES *and*
	“The hospital gave me a gift pack with formula”	NO
7. Enable mothers and their infants to remain together and to practice rooming-in 24 h a day.	“My baby stayed in the room with me at the hospital”	YES
8. Support mothers to recognize and respond to their infants’ cues for feeding.	“Hospital staff told me to breastfeed whenever my baby wanted”	YES
9. Counsel mothers on the use and risks of feeding bottles, artificial nipples (teats) and pacifiers.	“The hospital gave my baby a pacifier”	NO
10. Coordinate discharge so that parents and their infants have timely access to ongoing support and care.	“The hospital gave me a telephone number to call for help with breastfeeding”	YES

A summary variable was created to represent the full complement of the key clinical practices that mothers received. This variable was calculated by adding the total number of key clinical breastfeeding practices received during the hospital stay. It was determined that 13.9% of respondent mothers were missing information on receipt of at least one key clinical practice, and non-Hispanic White mothers were less likely to have missing data compared to other racial and ethnic groups (8.3% for non-Hispanic White mothers vs. 18.7% for other racial and ethnic groups). We decided against a complete case analysis due to missing data, and instead, we calculated the percent of steps mothers endorsed receiving. To be included in the calculation, respondents had to have non-missing data for at least five out of eight key clinical practices. Those that were missing data for more than three key clinical practices were excluded (*N* = 140). A categorical variable was created from the percent of key clinical practices received; 0–50%, 51–75%, 76–99%, and 100%. When a mother reported receiving 100% of key clinical practices, we considered this “ideal” breastfeeding care.

### Covariates

Potential demographic and clinical risk factors that were likely to be related to both race and ethnicity and receipt of key clinical practices were identified and controlled for in the analyses. Information on covariates from the birth certificate included maternal age, maternal education, medical insurance as Medicaid or non-Medicaid, mode of delivery as vaginal or c-section, parity as no child or 1 or more children, and infant birth weight as < 2500 or ≥ 2500 g.

### Statistical Analysis

Data were examined using SAS version 9.4 Survey Procedures and SAS-callable Statistical Software for Survey Data Analysis (SUDAAN Release 11.0) to account for the complex survey design of PRAMS data. We compared data from the 27 PRAMS sites from US states that collected information on BFHI key clinical practices to the full 44-site sample to identify variations in demographic characteristics and clinical variables that would affect generalizability. Descriptive analyses were performed to examine missing data, demographic and clinical characteristics and receipt of BFHI key clinical practices by race and ethnicity. Procedures used to examine the data included unweighted frequencies, weighted percents, 95% confidence intervals, and χ^2^ tests. We performed logistic regression to evaluate differences in receipt of individual BFHI key clinical practices by race and ethnicity using logistic regression and multinomial logistic regression for the nominal categorical variable representing the percent of BFHI key clinical practices a mother received. Analyses were performed with and without adjustment for clinical and demographic characteristics that were found to be significantly associated with maternal race and ethnicity in the descriptive analysis. We considered a two sided P value of less than 0.05 to be significant.

## Results

The 2016–2019 PRAMS survey sample included 161,702 respondent mothers. We excluded from our analyses 23,376 (14.5%) mothers who did not initiate breastfeeding, 22,228 (13.7%) who delivered infants before 37 weeks’ gestation, and 10,323 (6.4%) who were from Puerto Rico, had a multiple gestation, infant was not born in a hospital or was not living with mother at the time of the survey, or there was no available information on race and ethnicity (See Supplemental Fig. 1). The remaining sample included 105,775 respondent mothers; 60,395 mothers were from the 27 sites that included questions on hospital breastfeeding care. Respondent characteristics were similar in the 27-site sample compared to the full sample except for the region of residence in the US and the mother’s race and ethnicity. The 27-site sample included fewer mothers from the Midwest than the full sample, which resulted in a larger proportion of mothers identifying as Hispanic in the 27-site sample (21.1% in the 27-site sample vs. 19.2% in the full sample, see Supplemental Table 1).

Characteristics of respondent mothers and comparisons by race and ethnicity are listed in Table 2. Maternal age, maternal education, maternal Medicaid status, mode of delivery, parity, and infant birthweight were all significantly different among mothers across the racial and ethnic categories.

**Table 2:** Characteristics of PRAMS survey respondents, categorized by race and ethnicity

Characteristics	White NH		Black NH		Hisp-Eng		Hisp-Span		Asian/PI NH		AI/AN NH		Mix Race NH		
	N	%	N	%	N	%	N	%	N	%	N	%	N	%	*p* value
Total	28,032	56.2	8571	13.3	7871	11.6	4789	9.5	4851	6.0	3029	1.1	3252	2.3	
Maternal age															< 0.0001
<20 years old	782	2.8	516	5.9	726	9.8	227	5.3	41	0.8	286	9.0	226	6.6	
20–24 years old	4398	16.2	1978	23.8	2150	28.0	862	19.4	357	7.4	891	27.3	803	24.6	
25–29 years old	8555	29.9	2561	30.1	2420	27.9	1308	28.5	1263	27.5	938	31.9	976	29.2	
30–34 years old	9203	32.7	2105	23.6	1694	22.3	1288	25.8	1951	39.4	614	21.8	778	23.3	
35 + years old	5094	18.5	1411	16.6	881	12.0	1104	21.1	1239	24.9	300	9.9	469	16.3	
Maternal education															< 0.0001
<12 years	1293	4.9	799	9.9	1196	14.8	2355	47.4	300	7.8	575	17.4	285	8.5	
12 years	4917	18.4	2515	31.4	2337	31.5	1365	29.8	549	12.2	1099	35.9	775	24.5	
>12 years	21,691	76.3	5212	58.2	4304	53.3	993	21.7	3987	79.7	1339	46.4	2186	66.7	
Medical insurance															< 0.0001
Non-Medicaid	23,904	85.6	4947	62.8	5045	70.0	3691	81.8	4122	83.2	1580	54.2	2332	72.3	
Medicaid	4128	14.4	3624	37.2	2826	30.0	1098	18.2	729	16.8	1449	45.8	920	27.7	
Mode of delivery															< 0.0001
C-section	20,499	73.1	5699	67.2	5988	74.3	3402	70.7	3360	68.6	2349	75.7	2353	72.7	
Vaginal	7511	26.9	2870	32.8	1880	25.7	1387	29.3	1485	31.2	677	24.2	896	27.2	
Parity															< 0.0001
1	16,364	58.2	5304	61.7	4659	58.1	3543	71.5	2539	52.6	2032	66.8	1828	56.1	
2+	11,668	41.8	3267	38.3	3212	41.9	1246	28.5	2312	47.4	997	33.2	1424	43.9	
Infant Birthweight															< 0.0001
2,500 + g	26,053	98.2	7830	95.6	7440	97.3	4543	97.3	4517	96.4	2946	97.8	3111	97.3	
<2,500 g	1979	1.8	741	4.4	431	2.7	246	2.7	334	3.6	83	2.2	141	2.7	

Notes: Ns are unweighted. Percentages are weighted. Abbreviations: *NH* Non-Hispanic, *Hisp* Hispanic, *PI* Pacific Islander, *AI * American Indian, *AN* Alaska Native, *BF* Breastfeeding. Source: Pregnancy Risk Assessment Monitoring System (PRAMS) January 2016-December 2019

Receipt of key clinical practices varied by race and ethnicity, though some practices were widely but not universally reported, including receiving information about breastfeeding (Step 3), being provided information on breastfeeding from staff (Step 3), rooming-in (Step 7), and being encouraged to engage in cue-based infant-driven feeding (Step 8; Table 3). Only about half of mothers reported hospital staff not giving a pacifier (Step 9) during their hospital stay. About one-third of Black, Spanish-speaking Hispanic, and Asian/Pacific Islander mothers reported feeding only breastmilk in the hospital (Step 6), compared with more than half of White mothers.

**Table 3:** Maternal receipt of BFHI key clinical practices by maternal race and ethnicity, BFHI steps and percent of key clinical practices

Key clinical practices	White NH		Black NH		Hisp-English		Hisp-Spanish		Asian/PI-NH		AI/AN-NH		Mixed Race-NH		
	%	95% CI	%	95% CI	%	95% CI	%	95% CI	%	95% CI	%	95% CI	%	95% CI	*p* value
Step 3: Provided information on BF	96.0	(95.7, 96.3)	95.5	(94.8, 96.2)	95.0	(94.2, 95.9)	91.0	(89.7, 92.3)	96.4	(95.6, 97.1)	93.5	(91.5, 95.5)	95.9	(94.5, 97.3)	< 0.001
Step 4: STS and BF within 1st hour	80.7	(80.1, 81.3)	71.1	(69.5, 72.7)	75.1	(73.5, 76.8)	74.3	(72.3, 76.3)	70.0	(67.9, 72.2)	81.8	(78.9, 84.7)	77.9	(74.8, 80.9)	< 0.001
Step 5: Staff helped learn to BF	83.6	(83.0, 84.2)	86.1	(84.8, 87.4)	83.4	(81.8, 85.0)	83.4	(81.8, 85.0)	91.9	(90.7, 93.2)	79.1	(76.5, 81.6)	83.2	(80.6, 85.8)	< 0.001
Step 6: Feeding only breastmilk	54.7	(54.0, 55.5)	31.0	(29.3, 32.7)	40.8	(39.0, 42.7)	28.5	(26.5, 30.5)	31.9	(29.7, 34.1)	47.2	(44.0, 50.3)	48.0	(44.6, 51.4)	< 0.001
Step 7: Rooming in	93.3	(92.9, 93.7)	93.5	(92.6, 94.3)	93.8	(93.0, 94.6)	92.5	(91.4, 93.6)	90.0	(88.5, 91.5)	96.3	(95.5, 97.2)	93.2	(91.3, 95.2)	< 0.001
Step 8: Advised cue-based infant-driven feeding	90.0	(89.5, 90.5)	86.3	(85.1, 87.6)	85.7	(84.3, 87.1)	86.7	(85.2, 88.3)	89.1	(87.8, 90.4)	86.9	(84.2, 89.7)	86.1	(83.3, 88.9)	< 0.001
Step 9: Did not give pacifier	53.0	(52.2, 53.8)	46.8	(45.0, 48.7)	50.8	(48.9, 52.8)	51.0	(48.7, 53.3)	51.8	(49.4, 54.2)	45.9	(41.8, 50.0)	49.3	(45.5, 53.2)	< 0.001
Step 10: Phone # for lactation help after discharge	82.0	(81.4, 82.6)	80.4	(79.0, 81.9)	78.4	(76.8, 79.9)	65.5	(63.4, 67.7)	80.3	(78.5, 82.0)	76.5	(73.8, 79.1)	80.8	(77.9, 83.6)	< 0.001
Percent of key clinical practices															< 0.001
0–50%	10.4	(9.9, 10.9)	14.5	(13.3, 15.8)	13.6	(12.3, 15.0)	18.9	(17.2, 20.6)	14.0	(12.4, 15.6)	12.9	(10.4, 15.3)	13.0	(10.4, 15.8)	
51–75%	36.8	(36.0, 37.5)	48.7	(46.9, 50.5)	43.4	(41.5, 45.2)	47.1	(45.0, 49.3)	45.2	(42.8, 47.5)	37.8	(34.6, 41.0)	39.1	(35.8, 42.4)	
76–99%	29.8	(29.1, 30.5)	24.1	(22.5, 25.6)	26.5	(24.9, 28.1)	22.7	(21.0, 24.5)	26.8	(24.8, 28.7)	30.5	(27.9, 33.2)	27.6	(24.5, 30.8)	
100% (ideal care)	23.0	(22.4, 23.7)	12.6	(11.4, 13.9)	16.4	(15.1, 17.8)	11.2	(9.8, 12.6)	14.1	(12.3, 15.8)	18.8	(16.5, 21.1)	20.2	(17.7, 22.6)	

Notes: Ns are unweighted. Percentages are weighted. Abbreviations: *NH* non-Hispanic, *Hisp* Hispanic, *PI* Pacific Islander, *AI* American Indian, *AN* Alaska Native, *BF* Breastfeeding, *STS* Skin-to-skin. Source: Pregnancy Risk Assessment Monitoring System (PRAMS) January 2016-December 2019

Across all racial and ethnic categories, mothers were most likely to report receiving between 51 and 75% of key clinical practices (Table 3). Black non-Hispanic and Spanish-speaking Hispanic mothers were least likely to report receiving 100% of key clinical practices, while other racial and ethnic groups were least likely to receive 0–50% of practices. About one in four White non-Hispanic mothers reported receiving 100% of key clinical practices, “ideal” care, the highest proportion among any racial or ethnic group. Spanish-speaking Hispanic and Black non-Hispanic mothers were the least likely to receive “ideal” care, with rates about half of the level received by White mothers.

Multivariable analyses showed that after adjusting for maternal and infant characteristics, multiple individual key clinical practices in the hospital were less likely to be received by Black, Hispanic and Asian/Pacific Islander mothers compared to White non-Hispanic respondents, such as: skin-to-skin contact and breastfeeding in the first hour after birth (Step 4), feeding only breastmilk (Step 6), and being encouraged to engage in cue-based infant-driven feeding (Step 8; Table 4, see Supplementary Table 2 for the bivariate analyses). Few differences were noted by race and ethnicity in odds of being provided information on breastfeeding from staff (Step 3), rooming-in (Step 7), hospital staff not giving a pacifier (Step 9), and receiving a phone number to help with breastfeeding after discharge (Step 10), except Spanish-speaking Hispanic mothers who reported 32% reduced odds of being provided information on breastfeeding from staff (Step 3) and 43% reduced odds of receiving a phone number for lactation help after discharge (Step 10).

**Table 4:** Maternal receipt of BFHI key clinical practices by maternal race and ethnicity, compared to White non-hispanic women, adjusted for covariates

Key clinical practices	White NH	Black NH		Hisp-English		Hisp-Spanish		Asian/PI-NH		AI/AN-NH		Mixed Race-NH	
		OR	95% CI	OR	95% CI	OR	95% CI	OR	95% CI	OR	95% CI	OR	95% CI
Step 3: Provided information on BF	ref	1.05	(0.86, 1.28)	0.96	(0.78, 1.17)	0.58	(0.47, 0.71)	1.04	(0.82, 1.31)	0.81	(0.57, 1.17)	1.06	(0.74, 1.52)
Step 4: STS and BF within 1st hour	ref	0.67	(0.60, 0.73)	0.78	(0.70, 0.86)	0.76	(0.67, 0.86)	0.59	(0.52, 0.66)	1.14	(0.93, 1.40)	0.89	(0.74, 1.08)
Step 5: Staff helped learn to BF	ref	1.39	(1.23, 1.57)	1.37	(1.21, 1.54)	1.32	(1.14, 1.53)	2.14	(1.80, 2.55)	0.92	(0.78, 1.09)	0.99	(0.81, 1.21)
Step 6: Feeding only breastmilk	ref	0.44	(0.40, 0.48)	0.66	(0.61, 0.72)	0.44	(0.39, 0.49)	0.39	(0.35, 0.43)	0.90	(0.79, 1.04)	0.84	(0.73, 0.97)
Step 7: Rooming in	ref	1.16	(0.99, 1.36)	1.17	(0.99, 1.38)	0.90	(0.74, 1.09)	0.68	(0.57, 0.82)	2.02	(1.54, 2.64)	1.06	(0.77, 1.45)
Step 8: Advised cue-based infant-driven feeding	ref	0.82	(0.73, 0.93)	0.78	(0.68, 0.89)	0.91	(0.77, 1.07)	0.90	(0.69, 1.15)	0.76	(0.59, 0.96)	0.76	(0.59, 0.96)
Step 9: Did not give pacifier	ref	0.88	(0.80, 0.95)	1.03	(0.94, 1.12)	1.10	(0.99, 1.23)	0.94	(0.84, 1.04)	0.87	(0.73, 1.03)	0.92	(0.86, 0.98)
Step 10: Phone # for lactation help after discharge	ref	1.05	(0.94, 1.16)	0.95	(0.86, 1.06)	0.57	(0.51, 0.64)	0.87	(0.77, 0.99)	0.89	(0.76, 1.05)	1.02	(0.84, 1.23)

Abbreviations: *NH* Non-Hispanic, *Hisp* Hispanic, *PI* Pacific Islander, *AI * American Indian, *AN* Alaska Native, *BF* Breastfeeding. Source: Pregnancy Risk Assessment Monitoring System (PRAMS) January 2016-December 2019; Covariates: Maternal age, Maternal education, Medical insurance as Medicaid or non-Medicaid, Mode of delivery as vaginal or c-section, Parity as no child or 1 or more children, and Infant birth weight as < 2500 or ≥ 2500 g

Table 5 shows the results of the multivariable multinomial logistic regression analyses estimating the association between race and ethnicity and the percent of key clinical practices a mother received during her hospital stay (See Supplementary Table 3 for the bivariate analyses). Relative to White non-Hispanic respondent mothers, Black non-Hispanic, Hispanic, and Asian/Pacific Islander non-Hispanic mothers were significantly less likely to receive 100% of key clinical practices (ideal care) compared to every other category of percent of key clinical care practice. Overall, Black non-Hispanic mothers were 41% less likely to receive ideal care compared to White mothers, English-Speaking Hispanic respondents were 21% less likely, Spanish-speaking Hispanics were 37% less likely, and Asian/Pacific Islander non-Hispanic were 46% less likely, relative to White mothers. There were no significant differences found between American Indian/Alaska Native non-Hispanic and Mixed Race non-Hispanic mothers compared to White mothers.

**Table 5:** Percent of BFHI key clinical practices received by maternal race and ethnicity, compared to White non-hispanic women, adjusted for covariates

	Percent of Key Clinical Practices Received								
Race and Ethnicity	100% vs. 0–50%		100% vs. 51–75%		100% vs. 76–99%			100% (ideal care) vs. less	
	OR	95% CI	OR	95% CI	OR	95% CI		OR	95% CI
White NH	ref		ref		ref			ref	
Black NH	0.55	(0.47, 0.65)	0.52	(0.46, 0.59)	0.75	(0.65, 0.86)		0.59	(0.53, 0.67)
Hisp-English	0.74	(0.63, 0.86)	0.73	(0.66, 0.83)	0.88	(0.78, 0.99)		0.79	(0.71, 0.88)
Hisp-Spanish	0.46	(0.38, 0.57)	0.58	(0.49, 0.69)	0.81	(0.67, 0.96)		0.63	(0.53, 0.73)
Asian/PI-NH	0.44	(0.36, 0.54)	0.49	(0.41, 0.57)	0.68	(0.57, 0.80)		0.54	(0.47, 0.63)
AI/AN-NH	1.01	(0.78, 1.31)	1.08	(1.30, 0.88)	0.92	(0.77, 1.10)		1.01	(0.85, 1.19)
Mixed Race-NH	0.83	(0.64, 1.10)	0.93	(0.79, 1.12)	1.00	(0.82, 1.22)		0.95	(0.81, 1.11)

Abbreviations: *NH* Non-Hispanic, *Hisp* Hispanic, *PI * Pacific Islander, *AI * American Indian, *AN* Alaska Native, *BF* Breastfeeding. Source: Pregnancy Risk Assessment Monitoring System (PRAMS) January 2016-December 2019; Covariates: Maternal age, Maternal education, Medical insurance as Medicaid or non-Medicaid, Mode of delivery as vaginal or c-section, Parity as no child or 1 or more children, and Infant birth weight as < 2500 or ≥ 2500 g

## Discussion

This study used a population-based, nationally representative sample of mothers to study the association between race and ethnicity and receipt of BFHI key clinical practices in US hospitals. While some BFHI key clinical practices were experienced at high rates, practices were not experienced equally. Black non-Hispanic and Spanish-speaking Hispanic mothers received ideal care at almost half the rate of White non-Hispanic women. They also received fewer BFHI key clinical practices during their delivery hospital stay compared with White non-Hispanic women.

Rationales provided for health care disparities often focus on cultural, historical, social, psychosocial, political, and economic factors that contribute to inequities (Hemingway et al., 2021; Quintero et al., 2023). Rationales also focus primarily on individuals and health-related behaviors and and/or medical risk factors of different groups (Hailu et al., 2022). Yet, to address disparities in the *receipt* of health care we must look to health care workers and examine the impacts of structural racism, blind spots, and epistemic injustices that underlie the delivery of care (Monteiro et al., 2024).

Structural racism is deeply embedded in institutional practices, systems, and beliefs and is a fundamental driver of health inequities (Braveman et al., 2022; Cogburn, 2019). Racial and economic segregation have contributed to the development of hospitals that serve Black individuals and that often provide a lower quality of care (Hailu et al., 2022). Indeed, a decade ago, CDC data showed that facilities in zip code areas with higher percentage of Black residents were less likely to meet indicators for recommended practices supportive of breastfeeding (Lind et al., 2014). Since that time, in response to a call to address racial disparities in the US, Southern states with higher populations of minority people have seen an increase in the number of Baby Friendly Hospitals (Baby-Friendly, 2024). Yet, evidence of structural racism continues to be replete in studies in which researchers have shown that Black women are given less attention by health care providers (Sacks, 2019; Saluja & Bryant, 2021). Additionally, the underrepresentation of Black, Latino, and Native persons in health care professions and leadership positions continues to perpetuate the presence of structural racism (Lett et al., 2019; Salsberg et al., 2021).

Similarly, clinician blind spots in understanding are a result of limited lived experience, implicit or explicit indifferences, and devaluing of social or material elements (Johnson, 2020). For instance, people with access to transportation, childcare, and paid maternity leaves may devalue or ignore the absence of these resources as contributing factors when families make infant feeding choices. Instances of epistemic injustice occurs when the credibility or value of one individual is questioned by another (Percival et al., 2024). For example, a clinician may invalidate a woman’s concern about her infant’s hunger and associated requests for formula supplementation and view that mothers as someone who, “does not really want to breastfeed.” These various forms of racism promote a culture of oppression in which health workers may dismiss certain people as not deserving of care (Scott et al., 2024).

Results from our study are consistent with prior research on disparities in BFHI key clinical practices. Like previous studies, Black non-Hispanic women in our sample were less likely to initiate breastfeeding early and avoid pacifier use, however, we did not find differences in rates of rooming-in or receiving information on breastfeeding from hospital staff (Gee et al., 2012; Lind et al., 2014; Munn et al., 2018). Like Sebastian et al. (2019), we found that Hispanic women were more likely to use early formula supplementation, and Spanish-speaking Hispanic women were less likely to receive information on breastfeeding while in the hospital or a phone number to call for help after discharge. The finding that Spanish-speaking mothers received less health information corresponds with research that has shown that language-discordant health encounters result in reduced access to health information, worse outcomes, and decreased care satisfaction (Cano-Ibáñez et al., 2021; Diamond et al., 2019; Lor & Martinez, 2020). We found that Spanish-speaking mothers consistently received fewer key clinical practices than English-speaking mothers. This aligns with findings from prior researchers who have suggested it is inappropriate to treat Hispanic women as a homogenous group, since acculturation and nativity appear to be important predictors of breastfeeding outcomes (Bigman et al., 2018; Eilers et al., 2020).

Our study did not find differences in receipt of BFHI key clinical practices between American Indian/Alaska Native non-Hispanic mothers and White non-Hispanic mothers, a finding that is consistent with a prior study that included Native American non-Hispanic mothers from New Mexico (Sebastian et al., 2019). The lack of difference in BFHI key clinical practices could be explained by cultural practices among American Indian/Alaska Native non-Hispanic families that encourage breastfeeding (Secretary of Health and Human Services Advisory Committee on Infant and Maternal Mortality, 2022). It is also interesting to note that in 2011, the BFHI was brought to all Indian Health Service (IHS) hospitals by First Lady Michelle Obama’s “Let’s Move! In Indian Country” initiative to reduce childhood obesity (Indian Health Service, 2014).

This study has several limitations. First, as with any survey, bias is a concern. Survey respondents may differ from those who did not choose to respond. PRAMS addresses confounding due to response bias through weighting according to demographic data on the birth certificate, however, additional confounding may still be present. Recall bias is also an issue as the surveys were conducted at two to six months postpartum. It may not be possible to ascertain from these data if formula supplementation was due to medical need. To address this concern, we included only normal weight, full-term infants who were less likely to require formula supplementation. We noted that there were missing data for at least one of the survey items among 14% of respondents, and White non-Hispanic women had fewer missing data than women in other racial and ethnic groups. The most common missing question was related to pacifier use; the PRAMS question that addressed this issue may have been confusing to some respondents regarding whether hospital personnel gave the pacifier, or the mother requested it. We did not have information on the BFHI status of the hospitals where PRAMS mothers delivered their infants. Finally, we only included the care that women received and were not able to account for the care they requested. Mothers may have initiated the use of formula supplementation or a pacifier, and the care they requested could have been due to external factors, including cultural norms, lack of family support, or lack of access to paid maternity leave, among other reasons.

Racial and ethnic disparities exist in the receipt of clinical practices that support breastfeeding in US hospitals. Suboptimal breastfeeding care may be one of the barriers to breastfeeding that contributes to early breastfeeding cessation among women from certain racial and ethnic groups. Systems that establish and uphold national standards for equitable healthcare and develop key performance indicators for healthcare must be developed. More research is needed that focuses on practical strategies to dismantle systemic racism. Equitable care in the hospital is essential for all US families to have the best chance for breastfeeding success.

##  Supplementary Information

Below is the link to the electronic supplementary material.

Supplementary Material 1 (DOCX 104 KB)

## Data Availability

The data are available from the Pregnancy Risk Assessment Monitoring System (PRAMS) working group and the Centers for Disease Control and Prevention.

## Code Availability

Data cleaning and analysis code is available upon request from the corresponding author (JLT). The data sets generated and analyzed during this study are available from the corresponding author upon reasonable request. Data and metadata will be made available in the University of Rhode Island Research Data Repository (DigitalCommons @URI) after the completion of the project and for ten years.
