# Supplementary material for: Racial and Ethnic Disparities in Hospital Breastfeeding Care in the US
Source: Matern Child Health J. 2025 Jan 29;29(2):173–82. doi: 10.1007/s10995-025-04065-y (PMC11821785; doi:10.1007/s10995-025-04065-y)

Racial and Ethnic Disparities in Hospital Breastfeeding Care in the US

## **Supplemental Table 1:** Characteristics of respondents in 27-site and full samples across the study period

| Characteristics | 27-site sample | | | Full sample | | |
| --- | --- | --- | --- | --- | --- | --- |
|  | N | % | CI | N | % | CI |
| Total | 60,395 | 100 |  | 105,775 | 100 |  |
| Mat race and ethnicity |  |  |  |  |  |  |
| White NH | 28,032 | 56.2 | (55.7, 56.8) | 50,160 | 58.6 | (58.1, 60.0) |
| Black NH | 8,571 | 13.3 | (12.9, 13.7) | 17,216 | 13.3 | (13.0, 13.6) |
| Eng-speaking Hisp | 7,871 | 11.6 | (11.3, 12.0) | 12,123 | 10.7 | (10.4, 10.9) |
| Span-speaking Hisp | 4,789 | 9.5 | (9.1, 9.9) | 8,422 | 8.5 | (8.2, 8.7) |
| Asian/Pacific Islander NH | 4,851 | 6.0 | (5.7, 6.2) | 8,265 | 5.9 | (5.7, 6.1) |
| AI/AN NH | 3,029 | 1.1 | (1.0, 1.1) | 4,308 | 0.8 | (0.7, 0.8) |
| Mixed Race NH | 3,252 | 2.3 | (2.2, 2.5) | 5,281 | 2.4 | (2.2, 2.5) |
| Maternal age |  |  |  |  |  |  |
| <20 years old | 2,804 | 4.3 | (4.1, 4.6) | 4,527 | 4.0 | (3.8, 4.2) |
| 20-24 years old | 11,439 | 18.6 | (18.2, 19.1) | 18,755 | 17.9 | (17.5, 18.2) |
| 25-29 years old | 18,021 | 29.4 | (28.9, 30.0) | 31,333 | 29.4 | (29.0, 29.9) |
| 30-34 years old | 17,633 | 29.7 | (29.1, 30.2) | 32,086 | 30.5 | (30.1, 30.9) |
| 35+ years old | 10,498 | 18.0 | (17.5, 18.4) | 19,070 | 18.2 | (17.8, 18.5) |
| Maternal education |  |  |  |  |  |  |
| <12 years | 6,803 | 11.2 | (10.7, 11.6) | 11,577 | 10.4 | (10.1, 10.7) |
| 12 years | 13,557 | 22.7 | (22.1, 23.2) | 22,963 | 22.3 | (21.9, 22.7) |
| >12 years | 39,712 | 65.7 | (65.2, 66.3) | 70,521 | 66.7 | (66.3, 67.2) |
| Medical insurance |  |  |  |  |  |  |
| Non-Medicaid | 45,621 | 79.6 | (79.2, 80.1) | 79,949 | 79.2 | (78.9, 79.6) |
| Medicaid | 14,774 | 20.4 | (19.9, 20.8) | 25,826 | 20.8 | (20.4, 21.1) |
| Mode of delivery |  |  |  |  |  |  |
| Vaginal | 43,650 | 71.9 | (71.4, 72.5) | 75,903 | 71.8 | (71.4, 72.2) |
| C-Section | 16,706 | 28.0 | (27.4, 28.5) | 29,815 | 28.1 | (27.7, 28.6) |
| Parity |  |  |  |  |  |  |
| 0 | 36,269 | 59.6 | (59.0, 60.2) | 63,502 | 59.6 | (59.2, 60.1) |
| 1+ | 24,126 | 40.4 | (39.8, 41.0) | 42,273 | 40.4 | (39.9, 40.9) |
| Region of US |  |  |  |  |  |  |
| Midwest | 13,216 | 18.6 | (18.5, 18.7) | 28,225 | 26.2 | (26.2, 26.3) |
| Northeast | 14,119 | 25.8 | (25.7, 25.9) | 25,585 | 23.5 | (23.4, 23.6) |
| South | 14,587 | 39.4 | (39.3, 39.6) | 25,976 | 36.0 | (35.9, 36.1) |
| West | 18,473 | 16.2 | (16.1, 16.2) | 25,989 | 14.3 | (14.3, 14.4) |
| Infant characteristics |  |  |  |  |  |  |
| Birthweight |  |  |  |  |  |  |
| ≥2,500g | 56,440 | 97.5 | (97.4, 97.7) | 98,516 | 97.7 | (97.6, 97.7) |
| <2,500g | 3,955 | 2.5 | (2.3, 2.6) | 7,259 | 2.3 | (2.3, 2.4) |

Notes: 27 sites included questions about hospital practices that support breastfeeding. Ns are unweighted. Percentages are weighted. Abbreviations: NH = non-Hispanic; Hisp = Hispanic; PI = Pacific Islander; AI = American Indian; AN = Alaska Native. Source: Pregnancy Risk Assessment Monitoring System (PRAMS) January 2016-December 2019.

## Supplemental Table 2: Maternal receipt of BFHI key clinical practices by maternal race and ethnicity, compared to White non-Hispanic women, unadjusted

| Key Clinical Practices | White NH | Black NH | | Hisp-English | | Hisp-Spanish | | Asian/PI-NH | | AI/AN-NH | | Mixed Race-NH | |
| --- | --- | --- | --- | --- | --- | --- | --- | --- | --- | --- | --- | --- | --- |
|  |  | OR | 95% CI | OR | 95% CI | OR | 95% CI | OR | 95% CI | OR | 95% CI | OR | 95% CI |
| Step 3: Provided information | ref | 0.88 | (0.73, 1.07) | 0.79 | (0.65, 0.96) | 0.42 | (0.35, 0.50) | 1.10 | (0.87, 1.38) | 0.59 | (0.42, 0.83) | 0.97 | (0.68, 1.39) |
| Step 4: Initiate BF w/in 1^st^ hour | ref | 0.59 | (0.54, 0.64) | 0.72 | (0.66, 0.80) | 0.69 | (0.62, 0.77) | 0.56 | (0.50, 0.62) | 1.08 | (0.88, 1.31) | 0.84 | (0.70, 1.01) |
| Step 5: Helped learn | ref | 1.22 | (1.09, 1.36) | 1.25 | (1.11, 1.40) | 0.99 | (0.87, 1.11) | 2.24 | (1.89, 2.65) | 0.74 | (0.63, 0.87) | 0.97 | (0.80, 1.18) |
| Step 6: Only breastmilk | ref | 0.37 | (0.34, 0.40) | 0.57 | (0.53, 0.62) | 0.33 | (0.30, 0.37) | 0.39 | (0.35, 0.43) | 0.74 | (0.65, 0.84) | 0.76 | (0.66, 0.88) |
| Step 7: Rooming in | ref | 1.03 | (0.88, 1.20) | 1.09 | (0.93, 1.27) | 0.88 | (0.74, 1.05) | 0.65 | (0.54, 0.77) | 1.89 | (1.46, 2.45) | 0.99 | (0.73, 1.35) |
| Step 8: Advised BF on demand | ref | 0.70 | (0.62, 0.79) | 0.66 | (0.58, 0.75) | 0.73 | (0.63, 0.84) | 0.91 | (0.79, 1.05) | 0.74 | (0.58, 0.95) | 0.69 | (0.54, 0.88) |
| Step 9: Did not give pacifier | ref | 0.78 | (0.72, 0.85) | 0.92 | (0.84, 1.00) | 0.93 | (0.84, 1.02) | 0.95 | (0.86, 1.06) | 0.75 | (0.64, 0.89) | 0.86 | (0.74, 1.01) |
| Step 10: Given phone # for lactation | ref | 0.90 | (0.82, 1.00) | 0.80 | (0.72, 0.88) | 0.42 | (0.38, 0.46) | 0.90 | (0.79, 1.01) | 0.72 | (0.61, 0.83) | 0.92 | (0.77, 1.12) |

Notes: Abbreviations: NH = non-Hispanic; Hisp = Hispanic; PI = Pacific Islander; AI = American Indian; AN = Alaska Native; BF = breastfeeding. Source: Pregnancy Risk Assessment Monitoring System (PRAMS) January 2016-December 2019.

|  | Percent of Key Clinical Practices Received | | | | | | | | |
| --- | --- | --- | --- | --- | --- | --- | --- | --- | --- |
| Race and Ethnicity | 100% vs 0-50% | | 100% vs. 51-75% | | 100% vs. 76-99% | |  | 100% (ideal care) vs. less | |
|  | OR | 95% CI | OR | 95% CI | OR | 95% CI |  | OR | 95% CI |
| White NH | ref |  | ref |  | ref |  |  | ref |  |
| Black NH | 0.39 | (0.34, 0.45) | 0.41 | (0.37, 0.47) | 0.68 | (0.60, 0.78) |  | 0.48 | (0.43, 0.54) |
| Hisp-English | 0.55 | (0.47, 0.63) | 0.61 | (0.54, 0.68) | 0.80 | (0.71, 0.90) |  | 0.66 | (0.59, 0.73) |
| Hisp-Spanish | 0.27 | (0.22, 0.32) | 0.38 | (0.32, 0.44) | 0.64 | (0.54, 0.75) |  | 0.42 | (0.36, 0.49) |
| Asian/PI-NH | 0.45 | (0.37, 0.55) | 0.50 | (0.42, 0.58) | 0.68 | (0.57, 0.80) |  | 0.55 | (0.47, 0.63) |
| AI/AN-NH | 0.66 | (0.51, 0.85) | 0.79 | (0.66, 0.95) | 0.79 | (0.67, 0.94) |  | 0.77 | (0.66, 0.91) |
| Mixed Race-NH | 0.69 | (0.53, 0.91) | 0.83 | (0.69, 0.98) | 0.94 | (0.76, 1.15) |  | 0.84 | (0.72, 0.99) |

## Supplemental Table 3: Percent of BFHI key clinical practices received by maternal race and ethnicity, compared to White non-Hispanic women, unadjusted

Notes: Abbreviations: NH = non-Hispanic; Hisp = Hispanic; PI = Pacific Islander; AI = American Indian; AN = Alaska Native. Source: Pregnancy Risk Assessment Monitoring System (PRAMS) January 2016-December 2019.

Supplemental Figure 1: Flow chart of study population selection including inclusion and exclusion criteria


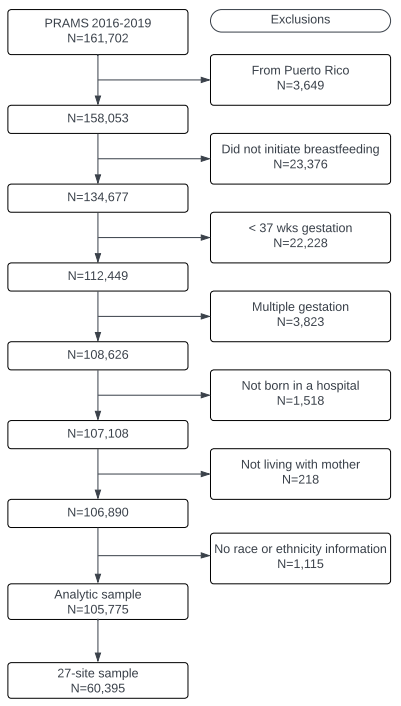

Supplement: Supplementary file 1 — Supplementary Material 1 (DOCX 104 KB) [file 10995_2025_4065_MOESM1_ESM.docx]
